# Knowledge, attitudes and practices relating to antibiotic use among community members of the Rupandehi District in Nepal

**DOI:** 10.1186/s12889-019-7924-5

**Published:** 2019-11-26

**Authors:** Anant Nepal, Delia Hendrie, Suzanne Robinson, Linda A. Selvey

**Affiliations:** 1 School of Public Health, Faculty of Health Sciences, Curtin University, Kent Street, Bentley, Perth, Western Australia 6102 Australia; 2 School of Public Health, The University of Queensland, Herston Rd, Herston, Qld 4006 Australia

**Keywords:** Antibiotic use, Antibiotic resistance, Knowledge, Attitudes, Practices, Nepal

## Abstract

**Background:**

The development of antibiotic resistance is one of the biggest threats to global public health. Inappropriate use of antibiotics is recognised as a leading cause of antibiotic resistance. The aim of this study was to explore the knowledge, attitudes and practices (KAP) towards antibiotic use among adults in Nepal.

**Methods:**

A quantitative survey was conducted with 220 community members of the Rupandehi district of Nepal, with cluster sampling techniques applied to select households. Interviews were carried out face-to-face using a structured questionnaire. Responses were presented using descriptive analysis, with chi-squared tests and regression analysis applied to identify factors associated with KAP about antibiotic use and the Spearman’s rank order correlation coefficient calculated to examine the relationship between responses to the KAP questions.

**Results:**

The sample comprised more females (54%) than males, the average age of respondents was 38.5 years and almost 60% of respondents lived in rural areas. Respondents had relatively good knowledge about aspects of antibiotic use other than identifying antibiotics. The concept of antibiotic resistance was well known but imperfectly understood. Half of respondents (50.9%) were unsure whether skipping doses would contribute to the development of antibiotic resistance, 88.2% indicated they would go to another doctor if not prescribed an antibiotic when they thought one was needed and nearly half (47.7%) believed antibiotics helped them get better more quickly if they had a fever. Most respondents reported correct practices accessing and using antibiotics, however, 84.6% at least sometimes preferred an antibiotic when they have a cough and sore throat.

Logistic regression showed respondents with higher levels of education tended to have better knowledge, more appropriate attitudes and better practices about antibiotic use. Rural respondents were less likely to have better knowledge about antibiotic use, while females were more likely to report better practices.

**Conclusion:**

The study provides baseline evidence about the knowledge, attitudes and practices regarding antibiotic use among the population of the Rupandehl district. Its findings will be useful in designing effective and targeted interventions to decrease misconceptions about antibiotic use and to increase awareness about the risks of inappropriate use of antibiotics in the community.

## Background

Inappropriate use of antimicrobial agents and the consequences of spread of antimicrobial resistance is an increasing public health problem [1]. In recent years, resistance to antimicrobial agents that were previously effective has emerged or re-emerged in many regions causing a global health threat and economic consequences. Among many other factors, behaviours of community members and their limited knowledge associated with inappropriate antibiotics use [2, 3] is contributing to antibiotic resistance. A recent review found one-third (33.7%) of the population of low and middle income countries lack knowledge about antibiotics and their role [4]. A study conducted in Bhutan found unsatisfactory knowledge (52.8%) and practices (47%) on antibiotic use [5]. Similarly, more than one-third (36%) of people in Kuwait reported not completing the prescribed course of antibiotics and around 28% had self-medicated with antibiotics [6].

A number of studies relating to antibiotic use in a range of different countries have investigated the knowledge, attitudes and practices of the general population [4, 7–16], secondary school teachers and university faculty members [17], students [18–21], primary care center attendants [22] and parents [23, 24]. These studies have shown patients’ or parents’ expectations of antibiotic therapy, or expectations as perceived by the doctor, to be a determining factor for antibiotic prescribing [6, 25, 26]. The rationale for educating the public is that knowledge about antibiotic treatment and awareness of antibiotic resistance are thought to influence patient and parent demand for antibiotic prescribing [27]. Because of wide cross-national differences in antibiotic use [28] tailoring of educational interventions requires determination of the needs of the audience in each country.

This paper reports on research that explores the knowledge, attitudes and practices of community members in relation to antibiotic use in the Rupandehi district in Nepal. Previous studies in Nepal have investigated surgical site infection and antibiotic use [29], antibiotic resistance [30–33], antibiotic prescribing and sensitivity [34], antibiotic prescribing patterns [35, 36], antibiotic dispensing practices [37], knowledge, attitudes and practices of medical students in relation to antibiotics use [38], and dispensing practices and patients’ knowledge about drug use [39]. However, to our knowledge no population based studies have been conducted on knowledge, attitudes and practices relating to antibiotics use. Moreover, some studies conducted in Nepal have found that antibiotics are among the most commonly sold drug classes [30, 37, 38]. Thus, it is important to measure this phenomenon, exploring the knowledge, attitudes and practices towards antibiotic usage, and awareness about anti-microbial resistance among adults of Nepal. The findings will aid in planning strategies for local health education purposes and developing intervention tools aimed at changing the practices of patients and the public.

## Methods

### Study area and sampling

A cross sectional quantitative survey of community members was conducted in the Rupandehi district of Nepal. At the time of developing the study design for this research, the administrative re-structure of Nepal had not been fully implemented. As per the earlier structure, Nepal was divided into five developmental regions and subdivided into 75 districts [40]. The districts were further divided into village development committees (VDCs) and municipalities, which were divided into wards as the basic administrative units. Districts are spread across three geographic regions, high-hill, hill and low-land, with approximately half of the population living in low-land regions [41].

According to the 2011 census, the total population of Nepal was 26,494,504 with 3.3% (880,196) of the population living in the Rupandehi district [41]. Almost two-thirds (66.0%, 580,688) were adults. The district is situated towards the central southern part of the country. As per the earlier structure, the district was divided into six municipalities and 42 VDCs. Municipalities and VDCs were aggregated in seven electoral areas [42].

We used guidelines developed by the World Health Organization (WHO) [43, 44] for the selection of households. Following these guidelines, public health facilities were selected as the basis of household selection. We chose six of the seven electoral areas to survey. Two were purposively selected: one that includes the largest hospital in the district and the other was the area with the lowest socio-economic status. An additional four areas were selected randomly from the remaining five electoral areas as recommended by the guidelines. One public health facility was selected from each survey area, consisting of all types of health facilities i.e. two each of hospitals (the largest as discussed above plus another), primary health care centres and health posts. The additional hospital plus other facilities were randomly selected. The VDCs and municipalities in which the public health facilities were located were used as the sampling area for selection of households. Of the total of six areas selected, four consisted of VDCs and two were municipalities.

A cluster sampling technique was applied to identify households to survey within the selected survey areas. Based on the WHO manual [45], we identified 20 clusters from the selected municipalities and VDCs. The smallest administrative unit, the “ward”, was considered as a cluster. Four clusters per municipality and three clusters per VDC were selected randomly. The sample size of 220 was based on an estimated prevalence of 33.7% of the population lacking knowledge on antibiotics and their role [4], a 95% confidence interval, a precision effect of 10%, a design effect of two to account for heterogeneity between clusters and an adjustment of 25% to allow for non-response [12, 46].

A list of households in each cluster was obtained from the records of respective municipalities and VDC offices. This list was verified after visiting each cluster and updated by deleting any duplicate households and adding any households missing from the records. Using the updated list of households in each cluster, an equal number of subjects (eleven) was selected from each cluster applying simple random sampling techniques.

The head of household was the preferred respondents for the study. However, if the head of household was absent at the time of interview, the most senior member of the household, who was 18 years and older, was interviewed.

### Data instrument and collection

A structured questionnaire was developed by adapting related questionnaires including one from the United States Agency for International Development (USAID) module “Antimicrobial resistance module for population-based surveys” [47] and those used in previous studies [48, 49] (Additional file 1).

A set of questionnaires was pre-tested with 30 respondents in urban and rural areas of the Nawalparasi district, Nepal (a neighboring district of Rupandehi), to ensure the cultural appropriateness, any problems with question wording, layout and understanding or a respondent’s reaction. As a result, minor adjustments were made to the final questionnaire based on the pre-test results. With a few people not knowing what the word “antibiotics” was, the questionnaire was amended to ask if they had heard of widely used antibiotics such as penicillin or metronidazole before being asked the main questions. Similarly a few respondents were unsure of the difference between “good” and “bad” bacteria present in our bodies so this difference was explained before they answered the question. Following explanation, issues with language did not appear to cause ambiguity that might impact on interpretation of the survey and the ensuing results. The final questionnaire included twelve questions relating to knowledge, eight questions to attitudes and six questions to practices. The reliability coefficient of responses to the final questionnaire was calculated using the Cronbach’s alpha score with the following results recorded: knowledge (0.63), attitudes (0.65) and practices (0.67).

The questionnaire comprised four sections: socio-demographic characteristics of respondents and a section on each of knowledge, attitudes and practices relating to antibiotics and their use. Questions about knowledge were divided into four domains, namely “identification of antibiotics” (Q1-Q3), “knowledge on the role of antibiotics” (Q4-Q6), “side-effects of antibiotics” (Q7-Q9) and “antibiotic resistance” (Q10-Q12). The questions on attitudes were divided into three domains: “preference for use of antibiotics” (Q13-Q15), “antibiotic resistance and safety” (Q16-Q18), and “attitudes to doctor’s prescribing of antibiotics” (Q19-Q20). The six questions relating to practices (Q21-Q26) were not divided into domains. The English version of the questionnaire was translated into Nepali and back translated into English to ensure the accuracy of the translated text.

Interviews were conducted in the Nepali language by two trained research assistants from September 2017 to December 2017. The training of research assistants covered the objectives of the study and familiarising them with the data collection techniques. A flow chart for the recruitment of respondents and consent process was provided to the research assistants and used in the data collection process. The average duration per interview was 20 min. Ten households were replaced in the original sample due to refusal to participate (*n* = 7) and no one at home at the time of interview (*n* = 3).

All respondents were informed of the nature of the study and written consent was sought to interviews being conducted. The study was approved by the Human Research Ethics Committee, Curtin University (HRE2017–0394) and the ethics committee of the Nepal Health Research Council (Reg. no.189/2017).

### Data management and analysis

Data were collected via paper-based questionnaires and the data were entered and analysed using the Statistical Package for the Social Sciences (SPSS) version 25.0 for Windows (IBM Corp., Armonk, NY, USA).

Demographic variables and responses to the knowledge, attitudes and practices questions were analysed using descriptive statistics. Responses to the five-point Likert scale for the knowledge and attitudes questions were combined into three groups: ‘strongly agree’ and ‘agree’, ‘strongly disagree’ and ‘disagree’, and ‘uncertain’. The three groups are referred to as “Yes”, “No” and “Don’t know”, respectively [50]. Questions relating to practices were assessed using the five-point Likert scales scoring scheme of ‘never’, ‘seldom’, ‘sometimes’, ‘often’ and ‘always’.

Regression analysis was conducted to identify demographic factors associated with knowledge, attitudes and practices. Responses to the knowledge and attitudes questions were given a score of “1” for a correct response and “0” for an incorrect or uncertain response, and scores summed for respondents across each of the domains. For the practices questions, responses were given a score based on the five-point Likert scale, ranging from “5” for the most appropriate answer to “1” for the least appropriate answer, and summed. The median score based on responses to questions in each of the knowledge, attitudes and practices sections was used as the cut-off to dichotomize the continuous variable for use as the dependent variable in multiple logistic regression analysis. Respondents scoring higher than the median were assessed as having “better knowledge”, “more appropriate attitudes” and “better practices” relating to antibiotic use [51]. The significance level (α) was set at 0.05 for all statistical tests. Spearman’s rank order correlation coefficient was used to describe the strength and direction of the relationship between responses to the knowledge, attitudes and practices questions.

## Results

### Characteristics of respondents

The sample consisted of 220 households (Table 1), with a response rate of 97% (*n* = 210). Compared to the adult population of the Rupandehi district, the sample included slightly more females (54% vs 52%) and respondents from rural areas (60% vs 51%). The mean age was 38.5 years (SD 11.5). Most respondents had achieved a level of education of primary/secondary school level (31.4%) or high school/intermediate level (30.0%). The mean monthly income of respondents was Nepalese Rupees (NPR) 42,491 (SD 16,835), compared with an estimated average monthly household income for Nepal of NPR 30,121 in 2015 [52].

**Table 1:** Socio-demographic characteristics of respondents

Variables	Study, n (% distribution)	Rupandehi, n (% distribution)^a^
Gender		
Male	101 (45.9)	277,714 (47.8)^b^
Female	119 (54.1)	302,974 (52.2)^b^
Area of residence		
Urban	88 (40.0)	(49.0)^b^
Rural	132 (60.0)	(51.0)^b^
Age (Years)		
15–24	25 (11.4)	185,430 (31.9)^b^
25–34	50 (22.7)	134,798 (23.2)^b^
35–44	81 (36.8)	99,013 (17.1)^b^
45–54	38 (17.3)	69,363 (11.9)^b^
55+	26 (11.8)	92,084 (15.9)^b^
Min = 18, Max = 69, Mean = 38.5, SD = 11.511		
Level of education		
General literate	38 (17.3)	
Primary/Secondary school	69 (31.4)	
High school/Intermediate	66 (30.0)	
Bachelors and above	47 (21.4)	
Level of Income - Quartile (NPR) (n = 210)		
First (30,000 or less)	60 (28.6)^c^	
Second (30,001 to 40,000)	46 (21.9)^c^	
Third (40,001 to 53,500)	52 (24.8)^c^	
Fourth (53,501 and above)	52 (24.8)^c^	
Min = 2300, Max = 110,000, Mean = 42,491.9, SD = 16,835.0		

^*a*^*Source: CBS, 2014* [53]

^*b*^*% calculated based on population of 15 years and above*

^*c*^*Numbers not equally distributed across quartiles due to clustering of responses at cut-off points*

### Knowledge, attitudes and practice relating to antibiotic use

**Fig. 1:**
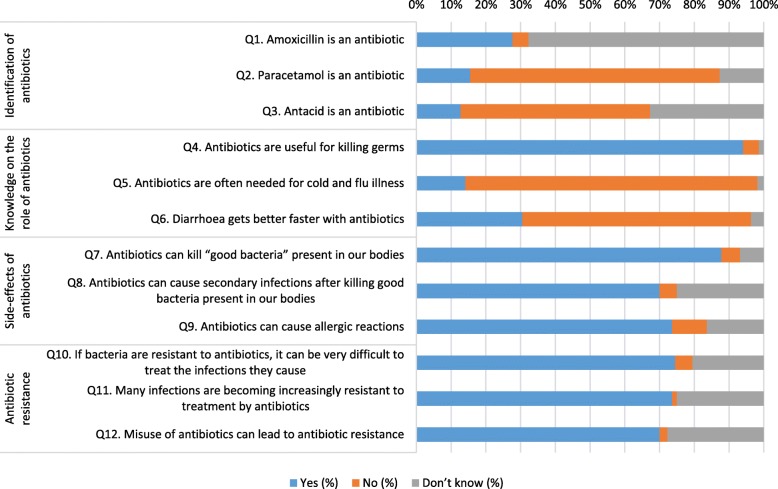
Responses to questions related to knowledge about antibiotic use

Respondents had relatively good knowledge about three of the four knowledge domains: “knowledge about the role of antibiotics (Q4-Q6)”, “side-effects of antibiotics (Q7-Q9)”, and “antibiotic resistance (Q10-Q12)” (Fig. 1). While the majority of responses to questions in the three domains were correct, for questions on “side-effects of antibiotics” and “antibiotic resistance” a relatively high percentage of responses to five of the six questions fell in the “don’t know” category (16–27%). Statements for which the “don’t know” response was highest included that “*antibiotics can cause secondary infections after killing good bacteria present in our bodies*” (25.0%), “*many infections becoming increasingly resistant to treatment by antibiotics*” (25.0%) and “*misuse of antibiotics leading to antibacterial resistance*” (27.7%).

Respondents had relatively less knowledge in regard to “identification of antibiotics (Q1-Q3)”. More than two-thirds (67.7%) did not answer correctly to the question that “*amoxicillin is an antibiotic*” and nearly one-third (32.7%) did not know that “*antacid is not an antibiotic*”. However, most respondents (94.1 and 84.1% respectively) answered correctly that “a*ntibiotics are useful for killing germs*” and “*antibiotics are not often needed for cold and flu illness*” while more than two-thirds (71.5%) knew paracetamol was not an antibiotic.

The level of knowledge about antibiotics use was better for respondents who lived in urban compared to rural areas (X^2^ = 16.257, *p* = < 0.001), for younger respondents (X^2^ = 30.696, *p* = < 0.001) and those with higher levels of education (X^2^ = 72.264, *p* = < 0.001) (Table 2).

**Table 2:** Responses to questions related to knowledge, attitudes and practices in relation to antibiotics use

Variables	Knowledge level			Attitudes level			Practices level		
	Less n (%)	Better n (%)	X^2^ (*p* = value)	Less appropriate n (%)	More appropriate n (%)	X^2^ (*p* = value)	Poor n (%)	Better n (%)	X^2^ (*p* = value)
Gender									
Male	55 (54.5)	46 (45.5)	0.010 (*p* = 0.920)	61 (60.4)	40 (39.6)	3.473 (*p* = 0.062)	76 (75.2)	25 (24.8)	5.984 (*p* = 0.014)
Female	64 (53.8)	55 (46.2)		86 (72.3)	33 (27.7)		71 (59.7)	48 (40.3)	
Area of Residence									
Urban	33 (37.5)	55 (62.5)	16.257 (*p* = < 0.001)	51 (58.0)	37 (42.0)	5.197 (*p* = 0.023)	46 (52.3)	42 (47.7)	13.996 (*p* = < 0.001)
Rural	86 (65.2)	46 (34.8)		96 (72.7)	36 (27.3)		101 (76.5)	31 (23.5)	
Age Group (Yr.)									
15–24	13 (52.0)	12 (48.0)	30.696 (*p* = < 0.001)	17 (68.0)	8 (32.0)	8.499 (*p* = 0.075)	10 (40.0)	15 (60.0)	17.921 (*p* = 0.001)
25–34	15 (30.0)	35 (70.0)		25 (50.0)	25 (50.0)		29 (58.0)	21 (42.0)	
35–44	40 (49.4)	41 (50.6)		58 (71.6)	23 (28.4)		55 (67.9)	26 (32.1)	
45–54	28 (73.7)	10 (26.3)		28 (73.7)	10 (26.3)		30 (78.9)	8 (21.1)	
55+	23 (88.5)	3 (11.5)		19 (73.1)	7 (26.9)		23 (88.5)	3 (11.5)	
Education Level									
General literate	36 (94.7)	2 (5.3)	72.264 (*p* = < 0.001)	33 (86.8)	5 (13.2)	27.306 (*p* = < 0.001)	31 (81.6)	7 (18.4)	42.452 (*p *= < 0.001)
Primary/Secondary School	51 (73.9)	18 (26.1)		53 (76.8)	16 (23.2)		55 (79.7)	14 (20.3)	
High School/Intermediate	25 (37.9)	41 (62.1)		43 (65.2)	23 (34.8)		48 (72.7)	18 (27.3)	
Bachelors and above	7 (14.9)	40 (85.1)		18 (38.3)	29 (61.7)		13 (27.7)	34 (72.3)	
Income Level - Quartile (NPR)									
First (30,000 or less)	36 (60.0)	24 40.0)	1.884 (*p* = 0.597)	44 (73.3)	16 (26.7)	1.512 (*p* = 0.680)	38 (63.3)	22 (36.7)	1.197 (*p* = 0.754)
Second (30,001 to 40,000)	24 (52.2)	22 (47.8)		30 (65.2)	16 (34.8)		29 (63.0)	17 (37.0)	
Third (40,001 to 53,500)	26 (50.0)	26 (50.0)		33 (63.5)	19 (36.5)		37 (71.2)	15 (28.8)	
Fourth (53,501 and above)	25 (48.1)	27 (51.9)		34 (65.4)	18 (34.6)		36 (69.2)	16 (30.8)	

**Fig. 2:**
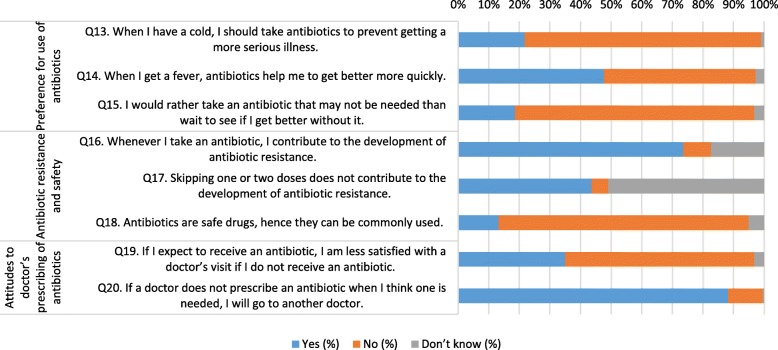
Responses to questions related to attitudes towards antibiotic use

Responses to questions about attitudes to antibiotics reflected varying points of view (Fig. 2). In terms of having a “preference for use of antibiotics (Q13-Q15)”, most respondents were aware that they did not need to take antibiotics for a cold to prevent them getting a more serious illness (77.3%) and did not want to take an antibiotic if they did not need one (78.2%). However, almost half (47.7%) thought antibiotics would help them to get better more quickly if they had a fever.

In the domain of “antibiotic resistance and safety (Q16-Q18)”, half of respondents (50.9%) were uncertain if skipping doses would not contribute to the development of antibiotic resistance and almost one fifth (17.3%) were uncertain about if taking an antibiotic contributed to the development of antibiotic resistance. Most respondents (81.8%) agreed antibiotics should not be commonly used.

Attitudes to prescribing of antibiotics by doctors were somewhat ambivalent. Most respondents (61.8%) were not less satisfied with a doctor’s visit if they did not receive an antibiotic; however, the majority (88.2%) indicated if they were not prescribed an antibiotic when they thought one was needed, they would go to another doctor.

Attitudes to antibiotic use was significantly associated with areas of residence (X^2^ = 5.197, *p* = 0.023) and education level (X^2^ = 27.306, *p* = < 0.001) (Table 2). Respondents living in urban areas and those with higher levels of education had more appropriate attitudes than those living in rural areas and those with lower levels of education.

**Fig. 3:**
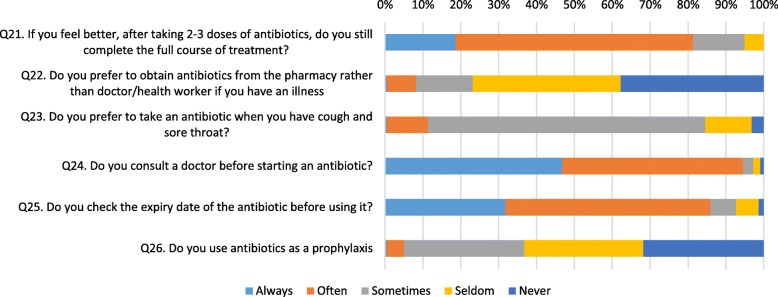
Responses to questions related to practices in relation to antibiotic use

In responding to questions about practices the majority always or often consulted a doctor before starting an antibiotic (94.5%), checked the expiry date of antibiotics before using them (85.8%) and completed the full course of treatment (81.3%), and never or seldom preferred to obtain antibiotics from the pharmacy (76.8%). However, in contrast to good practice reflected with these questions, the majority (84.6%) reported at least sometimes preferring to take an antibiotic when they have a cough or sore throat and almost a third (31.8%) to using antibiotics as a prophylaxis (Fig. 3).

Practices in relation to antibiotic use were significantly associated with gender (X^2^ = 5.984; *p* = 0.014), area of residence (X^2^ = 13.996, *p* = < 0.001), age group (X^2^ = 17.921, *p* = 0.001) and education level (X^2^ = 42.452; *p* = < 0.001) (Table 2). Female respondents, those who lived in urban areas, were younger and those with a higher level education reported better practices in regard to antibiotic use compared to males, respondents living in rural areas, older respondents and those with lower levels of education.

### Factors associated with knowledge, attitudes and practices relating to antibiotics use

In multivariable logistic regression analysis (Table 3), after adjusting for other variables, education level was found to be significantly associated with each of knowledge, attitudes and practices, with respondents with a level of education of Bachelor degrees and above having better knowledge, more appropriate attitudes and better practices. Area of residence was significantly associated with better knowledge on antibiotics use, with rural respondents being less likely to have better knowledge compared to urban residents, and females being more likely to have better practices than males.

**Table 3:** Odds ratios (ORs) of having better knowledge, more appropriate attitudes and better practices in relation to antibiotic use

Variables	Knowledge level			Attitudes level			Practices level		
	Crude OR^a^ (95% CI)	Adjusted OR^a^ (95% CI)	*p* = value	Crude OR^a^ (95% CI)	Adjusted OR^a^ (95% CI)	*p* = value	Crude OR^a^ (95% CI)	Adjusted OR^a^ (95% CI)	*p* = value
Gender									
Male	0.973 (0.572, 1.657)	0.867 (0.404, 1.859)	0.714	1.709 (0.971, 3.009)	1.672 (0.844, 3.311)	0.141	0.487 (0.272, 0.870)	0.394 (0.187, 0.829)	0.014
Female	1	1		1	1		1	1	
Area of Residence									
Rural	0.321 (0.183, 0.562)	0.317 (0.149, 0.676)	0.003	0.517 (0.292, 0.915)	0.587 (0.095, 1.492)	0.119	0.336 (0.188, 0.601)	0.553 (0.281, 1.085)	0.085
Urban	1	1		1	1		1	1	
Age Group (Yr.)									
15–24	3.621 (1.341, 9.777)	0.271 (0.060, 1.212)	0.088	1.301 (0.475, 3.561)	0.376 (0.95, 1.492)	0.164	7.227 (2.579, 20.254)	1.272 (0.313, 5.163)	0.736
25–34	9.154 (3.880, 21.595)	2.714 (0.861, 8.558)	0.088	2.765 (1.262, 6.057)	1.377 (0.480, 3.945)	0.552	3.489 (1.479, 8.233)	1.027 (0.324, 3.256)	0.964
35–44	4.021 (1.902, 8.502)	1.837 (0.741, 4.558)	0.189	1.096 (0.525, 2.288)	0.669 (0.278, 1.611)	0.370	2.278 (1.024, 5.067)	1.089 (0.430, 2.756)	0.858
44+	1	1		1	1		1	1	
Education Level									
Below secondary level	0.093 (0.042, 0.207)	0.035 (0.010, 0.127)	< 0.001	0.152 (0.071, 0.323)	0.147 (0.053, 0.411)	< 0.001	0.093 (0.042, 0.207)	0.098 (0.033, 0.293)	< 0.001
High school & intermediate	0.143 (0.062, 0.331)	0.285 (0.090, 0.906)	0.033	0.332 (0.153, 0.721)	0.337 (0.140, 0.812)	0.015	0.143 (0.062, 0.331)	0.155 (0.061, 0.939)	< 0.001
Bachelor degree and above	1	1		1	1		1	1	

*OR: Odds ratio.*

^*a*^*OR for included explanatory factors: adjusted with gender, residence, age and education*

Reference categories: 1 and better or more appropriate

*Respondents scoring higher than the median were assessed as having “better knowledge”, “more appropriate attitudes” and “better practices” relating to antibiotic use.*

### Relationship between knowledge, attitudes and practices

Spearman rank order correlation revealed a positive association between each pair of the knowledge, attitude and practice scores for respondents (*p* = < 0.001) (Table 4). The correlation was good between knowledge-attitudes and fair between knowledge-practices and attitudes-practices [54].

**Table 4:** Correlations between knowledge, attitudes, and practices

Variables	Correlation coefficient	*p*= value
Knowledge - Attitudes	0.649	< 0.001
Knowledge - Practices	0.428	< 0.001
Attitudes - Practices	0.370	< 0.001

However, comparing responses to questions in different domains, a few inconsistencies were noted. For example, although most respondents (84.1%) correctly answered the question in the knowledge section that antibiotics are not often needed for cold and flu illness (Fig. 1), in the practices section the majority (84.1%), answered that sometimes or often they preferred to take an antibiotic when they had a cough or sore throat (Fig. 3). Another example was respondents seemingly having good knowledge about antibiotic resistance (correct answers of between 70.0 to 74.5% for relevant questions) (Fig. 1), however more than one-third (35.0%) were less satisfied with a doctor’s visit if they did not receive an antibiotic and the majority (88.2%) would go to another doctor if a doctor did not prescribe an antibiotic when one was needed (Fig. 2).

## Discussion

This is the first study to identify the knowledge, attitudes and practices of the general population in Nepal regarding antibiotic use and to identify any factors associated with these main outcomes of interest.

Overall, the respondents in our study had relatively good knowledge about antibiotic use, with an exception being in regard to identification of antibiotics. More than two-thirds of respondents (67.7%) did not know that *“amoxicillin is an antibiotic”*, a significantly higher percentage than found in a study conducted in Bhutan (32.4%) [5]. Less than one-third of respondents (28.5%) did not know “*paracetamol is not an antibiotic”*, a similar result to that found in a Lebanese study (21.6%) [55]. We found that 15.9% of respondents were unsure whether *“antibiotics are often needed for cold and flu illness”*, a lower percentage than was found in studies conducted in Britain (42%) [11] and Lebanon (39%) [56].

With regard to attitudes towards antibiotic use, nearly half of respondents (47.7%) still believed that *“when they get a fever, antibiotics help them to get better more quickly”*, a comparable result to that found in a study conducted in Indonesia [14]. In the current study most respondents (88.2%) intended *“to go to another doctor if a doctor does not prescribe an antibiotic when they think one is needed”*. This suggests a high expectation in regard to using antibiotics for some illnesses or a low level of trust to prescribing practices of doctors. The latter was found in a Kuwaiti study, with one-third of respondents not trusting doctors who were not prescribing antibiotics [6].

Although respondents were aware that antibiotic resistance was a problem, half (50.9%) were still unsure whether “*skipping one or two doses does not contribute to the development of antibiotic resistance*”. This finding is consistent with a Palestinian study that found one-third of respondents knew the meaning of antibiotic resistance, however nearly one-third of them incorrectly agreed that antibiotics’ effectiveness would not be affected if antibiotics are taken less or more than the prescribed dose [57].

The only sociodemographic factor found to be associated with each of knowledge, attitudes and practices relating to antibiotics use was education. Respondents with higher education had better knowledge and more appropriate attitudes and practices, a finding consistent with other studies [5, 7, 13, 55, 57–60]. Our findings also suggest respondents in urban areas had better knowledge on antibiotic use than those in rural areas, a similar observation to that found in a Lithuanian study [61] but contrasting with a Polish study that found no such difference [62]. We found females to have better practices with regard to antibiotic use, a comparable result to a Hong Kong study [7].

A number of implications flow from our findings. Bringing about behavioural change is never easy, especially when it is deeply entrenched [63]. Our study provides an evidence base from which to develop education programmes for the community about antibiotic use. For example, given that several respondents failed to identify antibiotics, which could potentially risk antibiotics being used in a similar way to other drugs, educating the public on the roles of antibiotics and the ability to differentiate antibiotics from other drugs could help to minimise antibiotic misuse. The concept of antibiotic resistance is known but problems associated with antibiotic misuse were found to be imperfectly understood. The findings of the study also indicated that the community has high expectations with regard to being prescribed antibiotics, which increases the likelihood of non-prescription use of antibiotics. Village doctors or health workers could provide education to community members, and mass education campaigns conducted to emphasise the potential risks of resistance by using non-prescription antibiotics and the inappropriateness of using antibiotic therapy for minor ailments.

The study also identified a relationship between respondents having less knowledge, less appropriate attitudes and poor practices regarding the appropriate use of antibiotics. Groups, such as those with lower formal education, who had less knowledge and less appropriate attitudes and practices to antibiotic use and who could be targeted in education campaigns. A positive finding was females having better practices in regard to antibiotic use. In most developing countries, including Nepal, females hold the responsibility of taking care of their children and other family members, thus their better practices should contribute to some extent to the control of antimicrobial resistance.

Education of community members alone will not be enough to minimise any misuse of antibiotics. A multi-faceted approach involving policy makers, prescribers, and the general public using both educational and regulatory measures is needed. Such measures should be embedded in a general policy to change the culture of antibiotic use by improving awareness among the general public and professionals about the risks associated with antibiotic use as well as reducing public misconceptions about the benefit of taking antibiotics for minor illnesses.

This is the first known community survey conducted in Nepal to examine knowledge, attitudes and practices towards antibiotic use among the public. As such its findings provide baseline evidence about the knowledge, attitudes and practices regarding antibiotics use among the Nepalese population and offers insight in designing interventions to reduce antibiotic misuse.

The study used standard guidelines, developed by WHO [43, 44] for selecting the households. These guidelines have been refined continuously based on the lessons learned and used widely across many low and middle income countries to generate reliable information on medicines. Clustering and sampling techniques, described in the guidelines, are designed in a way to get optimum representation. Likewise, key aspects relating to antibiotic use in the community were covered by the survey questions, which were drawn from an USAID module on antimicrobial resistance [47] and previous studies [48, 49], which contributed to the validity of our study and allowed for comparison with previous results.

Another strength of the study was the high response rate (97%), which demonstrated representative results minimising the possible bias. However, the study was only conducted in one district in the low-land region of Nepal, so the results are more generalisable to districts in low-land regions. Also, surveys such as the one conducted in this study depend very much on the information given by respondents thus, the findings rely partly on the respondents’ honesty and ability to recall. Moreover, it is possible that respondents may over-report socially desirable behaviours or under-report socially undesirable behaviours. A limitation of the study was not using a social desirability scale to assess the extent of these behaviours. Additionally, the survey did not identify household structure and the knowledge, attitudes and practices might differ say among households with young children compared to those with older residents.

A possible problem in studies of antibiotic use involving lay people is whether the respondents know what antibiotics are. Respondents who had not heard the word “antibiotics” were asked if they had heard of widely used antibiotics such as penicillin or metronidazole before being asked the main questions. While nearly one-fifth (17%) of respondents did not understand the word “antibiotics”, all respondents were familiar with specific types of antibiotics following explanation by research assistants, thus to some extent combating this problem.

## Conclusion

This study is an important step towards a better understanding of the knowledge, attitudes and practices regarding antibiotic issues in the adult population in the Rupandehi district of Nepal. Its findings may be generalisable more broadly across the country, especially low-land regions. Our findings are important to the campaign to reduce the inappropriate use of antibiotics, and its findings can be used to inform the design of effective and targeted interventions to decrease misconceptions about antibiotic use and to increase awareness regarding the risks of inappropriate use. Its findings can also be used as a baseline for monitoring future interventions. Future studies should focus on the development and implementation of such public education measures to improve antibiotic use among community members in Nepal.

## Supplementary information

**Additional file 1.** Questionnaire.

## Data Availability

The datasets used and/or analysed during the current study are available from the corresponding author on reasonable request.

## Abbreviations

CI: Confidence Interval
KAP: Knowledge, Attitudes and Practices
NPR: Nepalese Rupees
OR: Odds Ratio
SPSS: Statistical Package for the Social Sciences
USAID: United States Agency for International Development
VDCs: Village Development Committees
WHO: World Health Organization
