# Supplementary material for: Knowledge, attitudes and practices relating to antibiotic use among community members of the Rupandehi District in Nepal
Source: BMC Public Health. 2019 Nov 26;19:1558. doi: 10.1186/s12889-019-7924-5 (PMC6880381; doi:10.1186/s12889-019-7924-5)
Supplement: Supplementary file 1 — Additional file 1. Questionnaire. [file 12889_2019_7924_MOESM1_ESM.docx]

# **Interview with Household Member: Knowledge, Attitudes and Practices of Antibiotic Use**

**Household Code: ______________**  **Interview Date: __________________**

**Municipality/Village Development Committee: _________________________ Ward No: _____**

**Section 1: Socio-Demographic**

| **Code** | **Name of Respondent** | **Relation to Head of Household** | | **Age** | **Sex** | **Education** |  |
| --- | --- | --- | --- | --- | --- | --- | --- |
| 101 |  |  | |  |  |  |  |
| **Note:**  **Sex:** 1. Male 2. Female  **Education:** 1. Illiterate, 2. Literate, 3. Primary/Secondary, 4. High School, 5. Intermediate, 6. Bachelors, 7. Masters, 8. PhD | | | | | | |  |
| 102 | What is your occupation, that is, what kind of work do you mainly do? | | …………………………………. | | | | |
| 103 | What is your type of family? | | Nuclear.………..……………………1  Joint…………….……………..……..2  Extended………………………….…3 | | | | |
| 104 | How many members are in your family? | | Total……………  Male……………  Female ……….. | | | | |
| 105 | What is your average monthly family income? | | Salary: NRs…………………..  Agriculture: NRs……………  Animal sales: NRs……………….  Interest: NRs……………..  Investments: NRs……………….  Business: NRs……………………  Others (Specify…………….): NRs……….  Don’t Know ………………………. | | | | |

**Section 2: Knowledge**

| **Code** | **Questions** | **Coding Category** |
| --- | --- | --- |
| 106 | Have you ever heard of a type of medicine called antibiotics? | Yes……………………………….......1  No …..……………………………......2 |

**Note:** If respondent says ‘No” please ask if they have heard of a widely used antibiotic such as penicillin or metronidazole before asking the questions from 107.

| **Codes** | **Domains** | **Questions** | **Response** | | | | |
| --- | --- | --- | --- | --- | --- | --- | --- |
|  |  |  | **Strongly Disagree** | **Disagree** | **Uncertain** | **Agree** | **Strongly Agree** |
| 107 | Identification of antibiotics | Amoxicillin is an antibiotic |  |  |  |  |  |
|  |  | Paracetamol is an antibiotic |  |  |  |  |  |
|  |  | Aluminium hydroxide+ Magnesium hydroxide (antacid) is an antibiotic |  |  |  |  |  |
| 108 | Knowledge on the role antibiotic | Antibiotics are useful for killing germs |  |  |  |  |  |
|  |  | Antibiotics are often needed for cold and flu illness |  |  |  |  |  |
|  |  | Diarrhoea gets better faster with antibiotics |  |  |  |  |  |
| 109 | Side-effects of antibiotics | Antibiotics can kill “good bacteria” present in our bodies |  |  |  |  |  |
|  |  | Antibiotics can cause secondary infections after killing good bacteria present in our bodies |  |  |  |  |  |
|  |  | Antibiotics can cause allergic reactions |  |  |  |  |  |
| 110 | Antibiotic resistance | If bacteria are resistant to antibiotics, it can be very difficult to treat the infections they cause |  |  |  |  |  |
|  |  | Many infections are becoming increasingly resistant to treatment by antibiotics |  |  |  |  |  |
|  |  | Misuse of antibiotics can lead to antibiotic resistance |  |  |  |  |  |

**Section 3: Attitudes and Practices**

**Section 3A**

| **Codes** | **Domains** | **Questions** | **Response** | | | | |
| --- | --- | --- | --- | --- | --- | --- | --- |
|  |  |  | **Strongly Disagree** | **Disagree** | **Uncertain** | **Agree** | **Strongly Agree** |
| 111 | Preference for use of antibiotics | When I have a cold, I should take antibiotics to prevent getting a more serious illness. |  |  |  |  |  |
|  |  | When I get a fever, antibiotics help me to get better more quickly. |  |  |  |  |  |
|  |  | I would rather take an antibiotic that may not be needed than wait to see if I get better without it. |  |  |  |  |  |
| 112 | Antibiotic resistance and safety | Whenever I take an antibiotic, I contribute to the development of antibiotic resistance. |  |  |  |  |  |
|  |  | Skipping one or two doses does not contribute to the development of antibiotic resistance. |  |  |  |  |  |
|  |  | Antibiotics are safe drugs, hence they can be commonly used. |  |  |  |  |  |
| 113 | Attitudes to doctor’s prescribing of antibiotics | If I expect to receive an antibiotic, I am less satisfied with a doctor’s visit if I do not receive an antibiotic. |  |  |  |  |  |
|  |  | If a doctor does not prescribe an antibiotic when I think one is needed, I will go to another doctor. |  |  |  |  |  |

# **Section 3B**

| **Codes** | **Questions** | **Response** | | | | |
| --- | --- | --- | --- | --- | --- | --- |
|  |  | **Almost always** | **Often** | **Sometimes** | **Seldom** | **Never** |
| 114 | If you feel better, after taking 2–3 doses of antibiotics, do you still complete full course of treatment? |  |  |  |  |  |
| 115 | Do you prefer to obtain antibiotics from the pharmacy rather than doctor/health worker if you have an illness |  |  |  |  |  |
| 116 | Do you prefer to take an antibiotic when you have cough and sore throat? |  |  |  |  |  |
| 117 | Do you consult a doctor before starting an antibiotic? |  |  |  |  |  |
| 118 | Do you check the expiry date of the antibiotic before using it? |  |  |  |  |  |
| 119 | Do you use antibiotics as a prophylaxis |  |  |  |  |  |

# **Note:** Questions are adopted from the following sources:

- *U.S. Agency for International Development, Management Sciences for Health, Macro International Inc. Antimicrobial resistance module for population-based surveys, Demographic and Health Survey. Washington, Arlington & Calverton: Management Sciences for Health & Macro International Inc, 2008.*
- *Huang SS, Rifas-Shiman SL, Kleinman K, Kotch J, Schiff N, Stille CJ, et al. Parental knowledge about antibiotic use: results of a cluster-randomized, multicommunity intervention. Pediatrics. 2007;119(4):698-706.*
- *Togoobaatar G, Ikeda N, Ali M, Sonomjamts M, Dashdemberel S, Mori R, et al. Survey of non-prescribed use of antibiotics for children in an urban community in Mongolia. Bulletin of the World Health Organization. 2010;88(12):930-6.*
